# Deletion of Murine APP Aggravates Tau and Amyloid Pathologies in the 5xFADXTg30 Alzheimer’s Disease Model

**DOI:** 10.3390/biom15020159

**Published:** 2025-01-21

**Authors:** Kunie Ando, Andreea-Claudia Kosa, Yasmina Mehadji, Hinde Lasri, Lidia Lopez-Gutierrez, Carolina Quintanilla-Sánchez, Emmanuel Aydin, Emilie Doeraene, Alain Wathelet-Depauw, Siranjeevi Nagaraj, Jean-Pierre Brion, Karelle Leroy

**Affiliations:** Alzheimer and Other Tauopathies Research Group, ULB Neuroscience Institute (UNI), ULB Center for Diabetes Research (UCDR), Faculty of Medicine, Université Libre de Bruxelles, 808 Route de Lennik, Bldg GE, 1070 Brussels, Belgium; andreea-claudia.kosa@ulb.be (A.-C.K.); hinde.lasri@ulb.be (H.L.); lidia.lopez.gutierrez@ulb.be (L.L.-G.); carolina.quintanilla.sanchez@ulb.be (C.Q.-S.); emmanuel.aydin@ulb.be (E.A.); emilie.doeraene@ulb.be (E.D.); alain.wathelet-depauw@ulb.be (A.W.-D.); siranjeevi.nagaraj@ulb.be (S.N.); jean-pierre.brion@ulb.be (J.-P.B.)

**Keywords:** Alzheimer’s disease, amyloid precursor protein, amyloid β, pTau, gliosis

## Abstract

Alzheimer’s disease is characterized by two key neuropathological lesions: amyloid plaques composed of amyloid β and neurofibrillary tangles formed by hyperphosphorylated tau. Amyloid β is produced through successive cleavages of amyloid precursor protein (APP) via the amyloidogenic pathway. While increasing evidence suggests that APP plays critical roles in neuronal function and that its proteolytic derivative, sAPPα, has neurotrophic effects, the impact of APP deletion on both amyloid and tau pathologies remains poorly understood. Here, we introduce a novel transgenic mouse model, 5xFAD×Tg30XAPP-/-, in which murine APP is deleted in the presence of both amyloid and tau pathologies. Using this innovative model, we demonstrate for the first time that deletion of APP exacerbates tau aggregation, amyloid deposition, and gliosis compared to control 5xFAD×Tg30 mice. This study provides the first in vivo evidence that APP deletion has profound and detrimental effects on both amyloid and tau pathologies in a transgenic model of Alzheimer’s disease, highlighting the previously unappreciated role of APP in the regulation of these neurodegenerative processes.

## 1. Introduction

Alzheimer’s disease (AD) is the most common form of dementia, characterized by a progressive cognitive decline and the presence of two key neuropathological lesions: amyloid plaques and neurofibrillary tangles (NFTs). In addition to these lesions, AD is consistently associated with a chronic immune response in the brain [1]. Activation of the brain’s resident astrocytes and macrophages (microglia) has been shown to exacerbate both amyloid and tau pathologies [1].

Amyloid plaques are composed of amyloid β (Aβ) peptides [2]. Aβ is produced through successive cleavages of amyloid precursor protein (APP) by β- and γ-secretases. Cleavage by β-secretase generates soluble N-terminal APPβ (sAPPβ) and membrane-bound β-C-terminal fragment (CTF). In contrast, in the physiologically predominant non-amyloidogenic pathway, APP is cleaved by α-secretase to produce soluble N-terminal APPα (sAPPα) and membrane-bound α-CTF [3]. sAPPα plays important roles in synaptogenesis, neurite outgrowth, neurogenesis, and cell adhesion [4,5]. Both α-CTF and β-CTF can be further cleaved by γ-secretase to produce p3 or Aβ, respectively, along with the amyloid precursor protein intracellular domain (AICD) [6]. Several mutations in the genes coding for *APP* and *presenilins* (*PS1*, *PS2*) increase Aß production and cause autosomal-dominant familial AD (FAD) [7].

NFTs are composed of paired-helical filament (PHF), which consists of hyperphosphorylated and aggregated microtubule-associated tau proteins [8]. While tau is natively unfolded, under pathological conditions, tau is hyperphosphorylated, detached from microtubules, misfolded, and mislocalized to the neuronal somatodendritic compartment. Hyperphosphorylated tau proteins then undergo oligomerization and fibrillization, eventually forming NFTs [9]. Tau pathology is also observed in related neurodegenerative diseases, including frontotemporal lobar degeneration with tau-immunoreactive inclusions (FTLD-tau), progressive supranuclear palsy (PSP), corticobasal degenerations (CBD), and others [9,10].

Many therapeutic developments in AD focus on targeting amyloid, including interventions aimed at various aspects of APP processing [11]. However, many of these therapies have been found to be largely ineffective or even detrimental [12], raising concerns about the risks associated with interfering in APP’s physiological functions. Further investigation into the consequences of disrupting or even abolishing APP functions is therefore needed to better understand the potential negative effects of therapies aimed at altering APP processing, particularly in the context of both amyloid and tau pathologies.

Previous studies have demonstrated that APP-deficient mice are viable and fertile but exhibit reduced locomotor activity and impaired forelimb grip strength [13,14]. When crossed with Tg30 tau transgenic mice, which overexpress human double-mutant tau, the resulting APPKOTg30 mice displayed an exacerbated neurological and muscular phenotype, accompanied by increased gliosis and tau pathology [15]. These findings consistently highlight the protective roles of endogenous murine APP and its derivatives. However, the impact of APP deletion on amyloid and tau pathologies in a transgenic model already exhibiting both amyloid and tau lesions has remained unclear.

In this study, we introduce a novel transgenic mouse line, 5xFADXTg30XAPP-/-, which combines three key genetic modifications: the 5xFAD line, which overexpresses human FAD-mutant APP and presenilin-1 (PS1) to model amyloid pathology [16]; the Tg30 line, which drives tau pathology through human tau overexpression [17,18]; and the APP-/- line [13]. This unique triple-transgenic model represents the first direct investigation of the consequences of complete murine APP deletion in the context of both amyloid and tau pathologies. Our findings reveal that 5xFADXTg30XAPP-/- mice exhibit significantly exacerbated motor deficits, more pronounced tau aggregation, increased amyloid deposits, and enhanced gliosis compared to control 5xFADXTg30 mice. These results underline the critical role of endogenous APP in modulating both amyloid and tau pathologies, providing new insights into the potential interplay between APP, tau, and amyloid in AD.

## 2. Materials and Methods

### 2.1. Mouse Lines

The 5xFAD double transgenic mice (Tg6799 line) were provided by Dr. Robert Vassar (Northwestern University, Chicago, IL, USA). These mice co-express and co-inherit the 695 amino acid isoform of human amyloid precursor protein (APP695) carrying the Swedish, Florida, and London mutations and the human PS1 with the M146L and L286V mutations [16]. Tg30 mice were provided by Dr. Luc Buée (University of Lille, Lille, France) and express the 1N4R human tau isoform with G272V and P301S mutations under the Thy1.2 promoter [17,18]. Only heterozygous 5xFAD, Tg30, and 5xFADXTg30 transgenic mice were used in this study. The APP-/- mouse line was purchased from Jackson Laboratories (Bar Harbor, ME, USA) and was generated using a targeting vector containing a neomycin resistance cassette and herpes simplex virus thymidine kinase genes to disrupt the promotor and exon 1 of the APP gene [13]. All the lines were maintained on a C57BL6J background.

5xFAD and Tg30 mice were crossed with APP-/- mice to generate F1 5xFADXAPP+/- and Tg30XAPP+/- mice. These were then crossed to generate F2 5xFADXTg30XAPP-/- mice, along with 5xFADXTg30XAPP+/+ (namely, 5xFADXTg30) mice in the same genetic background. For this study, only representative genotypes 5xFADXTg30 and 5xFADXTg30XAPP-/- were further analyzed. Genotyping was performed by two independent PCR amplifications of DNA extracted from tail samples, using primers for human mutant tau [18] and human APP [16]. The following primers were used to identify APP-/- mice: for the murine APP gene (sense primer 5′-AGAGCACCGGGAGCAGAG-3′, antisense primer 5′-AGCAGGAGCAGTGCCAAG-3′) and for the neomycin resistance sequence (sense primer 5′-CTTGGGTGGAGAGGCTATTC-3′, antisense primer 5′-AGGTGAGATGACAGGAGATC-3′).

Mice were sacrificed at 10 months by cervical dislocation without anesthesia, and brains were dissected. One hemisphere was snap-frozen in liquid nitrogen, while the other was fixed for 24 h in 10% formalin for histological analysis. All animal studies were performed in compliance with the ethical guidelines and approved by the Ethical Committee for the Care and Use of Laboratory Animals at the Medical School of the Free University of Brussels.

### 2.2. Rotarod Test

Motor deficits were assessed using a rotarod apparatus (Ugo Basile, Gemonio VA, Italy), as previously described [18]. Briefly, animals underwent training sessions consisting of three consecutive trials per day over three days. During each trial, animals were placed on a rotating rod with a progressive acceleration from 4 to 40 rpm. The day before testing, animals were individually housed to ensure they were acclimated to the testing environment. On the test day, animals were evaluated for a maximum of 300 s. The latency to fall off the rotarod was recorded as the mean of the three trials performed on the third day. If an animal stayed on the rotarod for the full 300 s, it was removed, and the latency was recorded as 300 s.

### 2.3. Wire Hang Test

The wire hang test was performed by placing mice on top of a cage lid, as previously described [19]. This lid was then inverted to a height of 20 cm above the cage litter for a maximum of 60 s. This test was conducted over three consecutive trials across three days. The latency to fall off the wire was recorded for each of the nine sessions, and the mean latency was calculated.

### 2.4. Antibodies

The rabbit polyclonal BR15 antibody was raised against a synthetic peptide corresponding to the last 20 amino acids of human APP and conjugated to purified protein derivative, and it reacts with both mouse and human APP [20]. The mouse monoclonal anti-APP antibody 3H5 is specific for human APP [21]. The rabbit polyclonal BR21 antibody was raised against human tau by immunizing rabbits with the synthetic peptide C-GTYGLGDRKDQGG and is specific for human tau [22]. Rabbit polyclonal anti-tau (A0024), purchased from Dako, reacts with both mouse and human tau. Mouse monoclonal antibodies against β-actin (A5441), β-tubulin (T4026), and Glial Fibrillary Acidic Protein (GFAP, clone GA5, catalog number C9205) were purchased from Sigma-Merck. The mouse monoclonal anti-pTau (Ser202 and Thr205) AT8 antibody (MN1020) was purchased from ThermoFisher. The mouse monoclonal PHF1 and MC1 antibodies were provided by Dr. Peter Davies. PHF1 recognizes tau phosphorylated at Ser396/404 [23], and MC1 reacts with tau in a pathological conformation requiring both an N-terminal fragment and a C-terminal fragment [24]. Mouse monoclonal anti-Aβ (4G8) antibody was purchased from Covance. Goat polyclonal anti-Iba-1 antibody (ab5036) was purchased from Abcam.

### 2.5. Preparation of Brain Homogenates for Biochemical Analysis

Mouse brain hemispheres were homogenized, as previously described [22,25,26] (Figure S1), in 10 volumes of ice-cold RIPA buffer containing 50 mM Tris-HCl (pH 7.4), 50 mM NaCl, 1% NP-40, 0.25% sodium deoxycholate, 5 mM EDTA, 1 mM EGTA, complete protease inhibitor cocktail (Merck, Darmstadt, Germany, 11836170001), 1 mM PMSF (Sigma, St. Louis, MO, USA, P-7626), and phosphatase inhibitor cocktail 2 (Sigma, P-5726) and incubated for 60 min at 4 °C on a rotator. The total homogenate was centrifuged (20,000× *g* for 20 min at 4 °C), and the supernatant was collected as the RIPA-soluble fraction. Protein concentrations were determined by the Bradford method (Bio-Rad, Hercules, CA, USA, 5000205) before the addition of Laemmli sample buffer. Twenty-five micrograms of protein were loaded to each well for SDS-PAGE.

### 2.6. Analysis of Sarkosyl-Insoluble PHF-Tau Fraction

One milliliter of the RIPA-soluble fraction was subjected to sarkosyl fractionation by incubating with 1% (*wt.*/*v*) N-lauroylsarcosine (Sigma-Aldrich, Merck, L-5125) under mild rotation for 30 min at room temperature (RT) and then centrifuged for 30 min at 180,000× *g* at 4 °C, as previously described [25,27,28,29,30]. The pellet, containing the sarkosyl-insoluble material after ultracentrifugation, was gently rinsed with 500 µL of phosphate-buffered saline (PBS) and suspended in 100 µL of PBS by rigorous vortexing and pipetting. The total protein concentration was measured by the Bradford protein quantification method (Bio-Rad 5000001). The sarkosyl-insoluble fraction was analyzed by Western blot (WB).

### 2.7. WB

Tissue lysates were separated by SDS-PAGE on 7.5% or 10% of Tris-Glycine gels and transferred to nitrocellulose membranes (sc-3724, Santa Cruz Biotechnology, Dallas, TX, USA). The nitrocellulose membranes were blocked in 10% (*w*/*v*) semi-skimmed dry milk in tris-buffered saline (TBS) (Tris-HCl 0.01 M, NaCl 0.15 M, pH 7.4) for 1 h at room temperature, then incubated with primary antibodies overnight. After several rinses, the membranes were incubated with either anti-rabbit (#7074, Cell Signaling Technology, Danvers, MA, USA) or anti-mouse (A-6782, Sigma) immunoglobulin antibodies conjugated to horseradish peroxidase. Following additional rinses, the membranes were incubated with SuperSignal West Pico PLUS Chemiluminescent Substrate (Pierce, Appleton, WI, USA) and exposed to a DARQ-7 CCD cooled camera (Vilber-Lourmat) in a SOLO 4S WL system. The optical density (OD) of protein signals was quantified by densitometric analysis using NIH ImageJ software (version 1.53a). The OD of the actin signal was used for normalization of protein loading in the RIPA-soluble fraction. For sarkosyl-insoluble fraction, the OD was normalized to the total protein concentration.

### 2.8. Histological Staining and Immunocytochemistry

Mouse brain hemispheres were fixed in 10% formalin for 24 h before being embedded in paraffin. Seven-micrometer-thick brain sections were mounted onto SuperFrost Plus slides (VWR) and dried at 37 °C. Immunohistochemical labeling was performed using the ABC method (Elite), as previously described [29]. Briefly, deparaffinized tissue sections were treated with H_2_O_2_ to inhibit endogenous peroxidase activity and incubated with a blocking solution of 10% (*v*/*v*) normal horse serum in TBS (0.01 M Tris, 0.15 M NaCl, pH 7.4). After overnight incubation with the diluted primary antibody, the sections were sequentially incubated with either horse anti-mouse or goat anti-rabbit antibodies conjugated to biotin (Vector), followed by the ABC complex (Vector). Peroxidase activity was developed using diaminobenzidine (Dako) as chromogen.

### 2.9. Quantitative Analyses of Histological Staining

Quantitative analysis of immunolabelling for tau, Aβ, and glial markers (GFAP and Iba-1) was performed using NIH ImageJ software (version 1.53a). Analyses were conducted on sagittal brain sections at a lateral level approximately 1 mm from the midline (Allen Brain Atlas) with a 4× objective on a Leica DM500 microscope, as previously reported [15,31]. The percentage of area occupied by immunolabeling was calculated by measuring the labeled area within the hippocampus and dividing it by the total surface area of the hippocampus. For tau staining analyses of MC1, PHF1 and AT8 were performed by counting pTau-positive neurons using NIH ImageJ Cell Counter tool. The number of pTau-positive neurons in the hippocampus was divided by the total surface area analyzed.

### 2.10. Statistical Analyses

The number of samples is indicated in the figure legends. Statistical analyses and normality tests were performed using GraphPad Prism 9. Comparisons were conducted using unpaired two-tailed Student’s *t*-tests for parametric data, Mann–Whitney U tests for non-parametric data, or one-way ANOVA, as specified in the figure legends. Data are presented as mean ± SEM (standard error of the mean). *P* values < 0.05 were considered statistically significant.

## 3. Results

### 3.1. Expression of APP and Tau Proteins in 5xFADXTg30 and 5xFADXTg30XAPP-/- Mouse Brains

**Figure 1:**
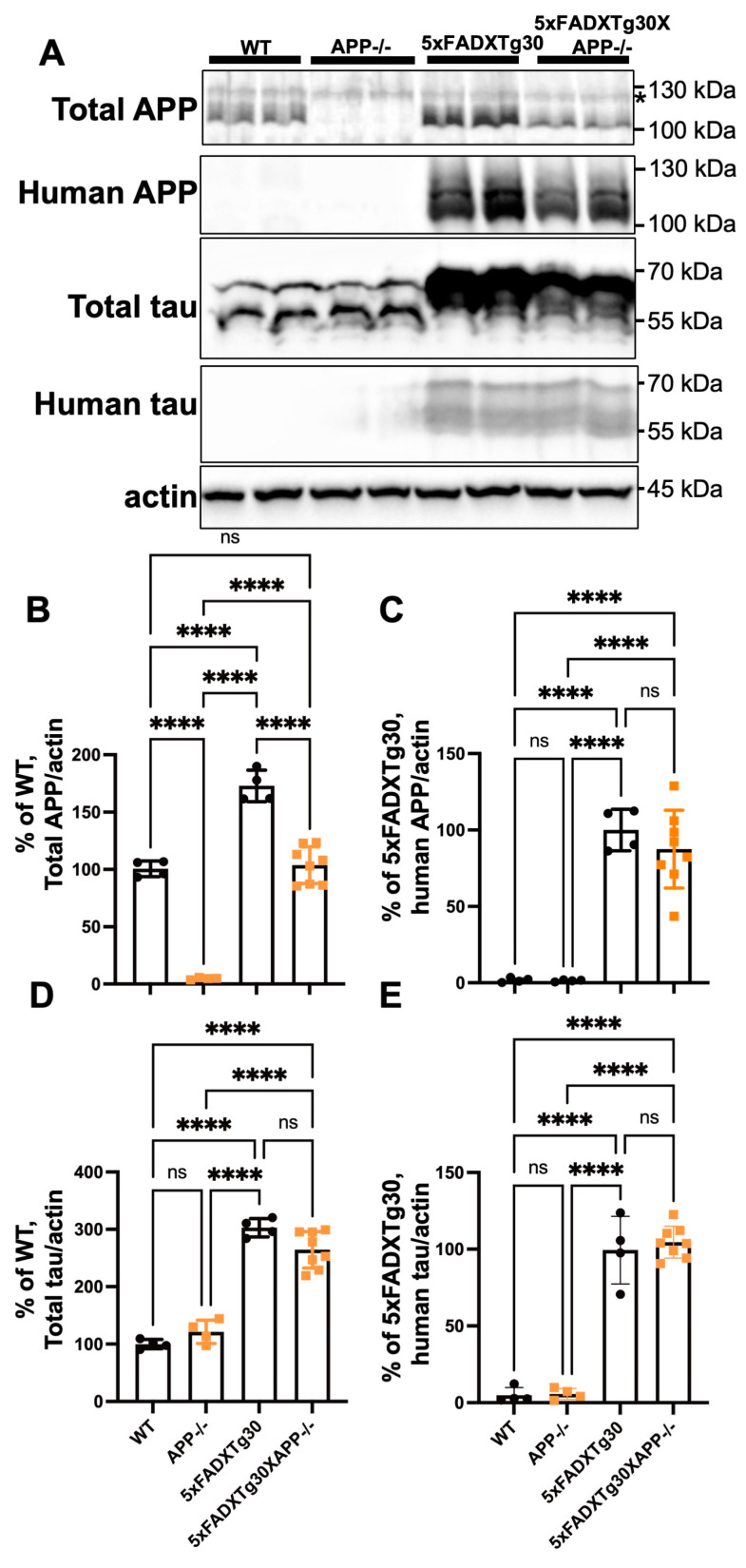
Total APP levels are reduced in the brains of 5xFADXTg30XAPP-/- mice compared to 5xFADXTg30 mice. (**A**) Representative WB showing total APP (BR15), human APP (3H5), total tau (A0024), human tau (BR21), and actin in the RIPA-soluble fraction of male mouse brains at 10 months. A non-specific band detected by anti-APP (BR15) antibody is indicated by an asterisk. (**B**–**E**) Quantification of total APP detected by BR15 (**B**), human APP detected by 3H5 (**C**), total tau detected by A0024 (**D**), and human tau detected by BR21 (**E**), all normalized to actin in brain lysates from WT, APP-/-, 5xFADXTg30, and 5xFADXTg30XAPP-/- mice (n = 4, n = 4, n = 4, and n = 8, respectively). Data are presented as mean ± SEM (* *p* < 0.05, **** *p* < 0.0001 by one-way ANOVA with Tukey’s post hoc test). The original blots are shown in Figures S2–S5.

We first analyzed the expression levels of APP and tau in the RIPA-soluble fraction of 10-month-old male WT, APP-/-, 5xFADXTg30, and 5xFADXTg30XAPP-/- mouse brains (Figure 1A and Figures S2–S5). WB analyses confirmed that APP-/- mice showed no specific band for total APP (murine and human) around 100–130 kDa. In contrast, in transgenic mice bearing the 5xFAD genes, human APP expression was detected using the 3H5 antibody, resulting in significantly increased total APP (murine and human) expression in the brains of 5xFADXTg30 mice. In the brains of 5xFADXTg30XAPP-/- mice, APP expression was reduced due to the deletion of murine APP, compared to 5xFADXTg30 mice (Figure 1B). However, the level of human APP was not significantly altered between the brains of 5xFADXTg30 and 5xFADXTg30XAPP-/- mice (Figure 1C), with variabilities observed among individual samples (Figure S3). In both 5xFADXTg30 and 5xFADXTg30×APP-/- brains, total tau expression was increased compared to WT or APP-/- controls, likely due to the expression of human mutant tau. The level of tau expression detected in the RIPA-soluble fraction was similar in 5xFADXTg30 and 5xFADXTg30XAPP-/- mice for total (murine and human) tau (Figure 1D) and human tau (Figure 1E). These data suggest that while total APP expression was reduced in 5xFADXTg30XAPP-/- due to the deletion of murine APP, total tau level remained similar between 5xFADXTg30 and 5xFADXTg30XAPP-/- mice.

### 3.2. Murine APP Deletion in 5xFADXTg30 Mouse Model Aggravates Muscle Weakness and Motor Phenotype

**Figure 2:**
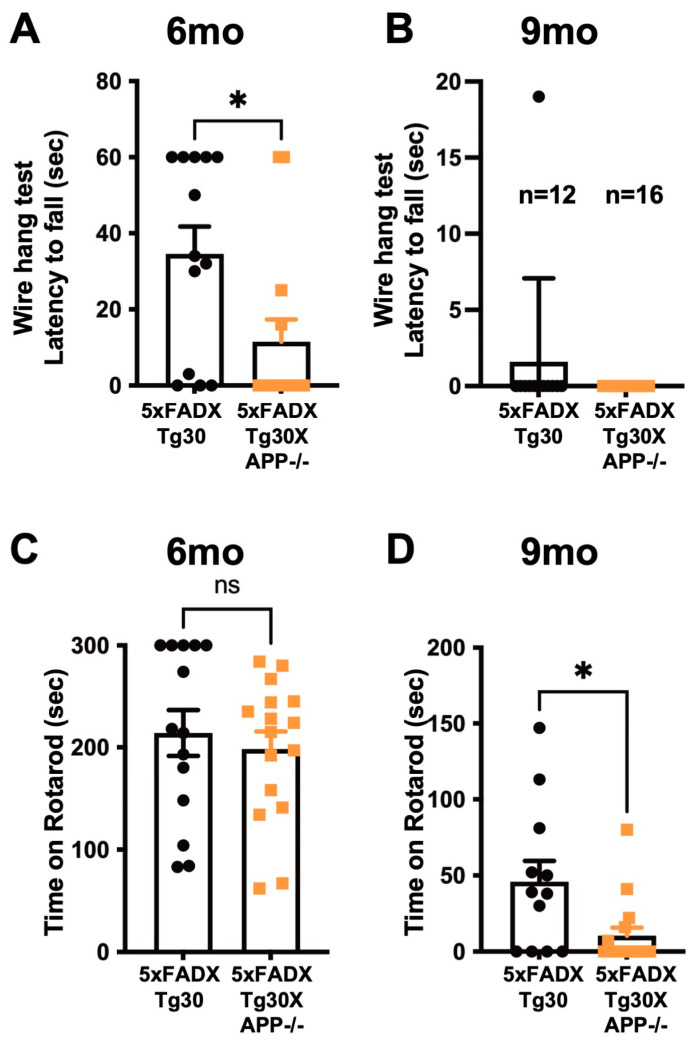
Motor phenotypes are aggravated in 5xFADXTg30XAPP-/- mice compared to 5xFADXTg30 mice. (**A**,**B**) Performance on the wire hang test is shown as the latency to fall from a suspended grid. At 6 months, 5xFADXTg30XAPP-/- mice exhibited a significantly reduced latency compared to 5xFADXTg30 mice (n = 13 for 5xFADXTg30 and n = 14 for 5xFADXTg30XAPP-/-). (**B**) By 9 months, mice displayed severe muscle weakness, with most achieving a score of 0 s, except for one 5xFADXTg30 mouse. No significant difference in latency was observed between 5xFADXTg30 and 5xFADXTg30XAPP-/- mice (n = 12 and n = 16, respectively). (**C**,**D**) Performance on the accelerated rotarod is shown. (**C**) At 6 months, there was no significant difference in latency between 5xFADXTg30 and 5xFADXTg30XAPP-/- mice (n = 14 and n = 16, respectively). (**D**) At 9 months, 5xFADXTg30XAPP-/- mice had a significantly reduced latency on the rotating rotarod compared to 5xFADXTg30 mice (n = 12 and n = 16, respectively). Data are presented as mean ± SEM (* *p* < 0.05 by Mann–Whitney U test).

Previous studies have shown that APP-/- mice exhibit decreased locomotor activity and muscle weakness [13,14]. Consistently, APP deletion exacerbated the motor phenotype and muscle weakness in APPKOTg30 mice compared to Tg30 controls [15]. To analyze the effect of murine APP deletion on the phenotype of 5xFADXTg30 mice, which model both amyloid and tau pathologies [32], muscle strength was assessed using the wire hang test at 6 and 9 months of age (Figure 2A,B). At 6 months, 5xFADXTg30XAPP-/- mice showed significantly aggravated muscle weakness compared to 5xFADXTg30 controls (Figure 2A). At 9 months, both genotypes exhibited severe muscle weakness and reduced latency on the wire, with no significant difference observed between the two groups (Figure 2B). The mice were also analyzed with the rotarod test at 6 and 9 months (Figure 2C,D). While no significant difference was observed at 6 months (Figure 2C), at 9 months, 5xFADXTg30XAPP-/- mice had significantly reduced time on the accelerating rotarod compared to 5xFADXTg30 mice (Figure 2D). We also compared the motor phenotype between WT and APP-/- mice at 6 and 9 months. However, no significant differences were observed between WT versus APP-/- in our experimental condition (Figure S6A–D). Taken together, these data suggest that the deletion of murine APP in the 5xFADXTg30 mouse model exacerbates muscle weakness and motor deficits.

### 3.3. Tau Pathology Is Aggravated in 5xFADXTg30XAPP-/- Mouse Brains Compared to 5xFADXTg30

**Figure 3:**
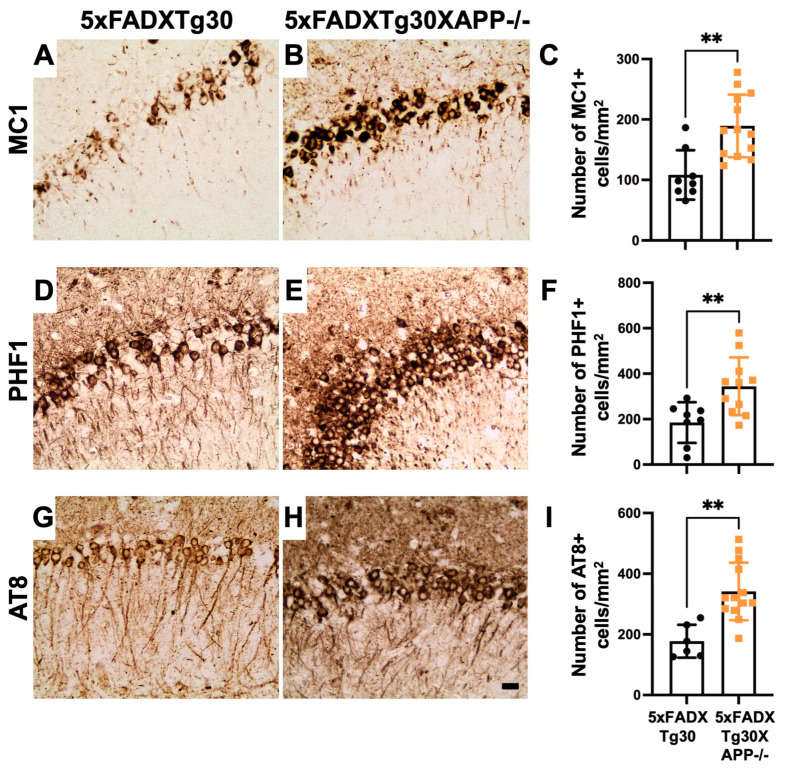
Murine APP deletion enhances tau pathology progression in the hippocampus of 5xFADXTg30XAPP-/- mice compared to 5xFADXTg30. (**A**–**I**) Representative immunostaining for pathological tau using MC1 (**A**,**B**), PHF1 (**D**,**E**), and AT8 antibodies (**G**,**H**) in the CA1 pyramidal neurons of the Ammon’s horn of the hippocampus from 5xFADXTg30 and 5xFADXTg30XAPP-/- mice. There was a significant increase in the number of neurons immunolabelled for misfolded tau (MC1) and hyperphosphorylated tau (pSer396/Ser404 for PHF1 and pSer202/Thr205 for AT8) in 5xFADXTg30XAPP-/- mice compared to 5xFADXTg30 mice (**C**,**F**,**I**). Analysis was performed on 10-month-old male mice (5XFADXTg30: n = 6–8 and 5xFADXTg30XAPP-/-: n = 11–13). Data are presented as mean ± SEM (** *p* < 0.01 by unpaired *t*-tests). Scale bar, 20 µm.

To determine whether murine APP deletion affects tau pathology progression in the 5xFADXTg30 model, we assessed tau pathology by immunohistochemistry using antibodies that detect tau in pathological conformation (MC1), pSer396/Ser404 tau (PHF1), and pSer202/Thr205 tau (AT8) (Figure 3). pTau-positive neurons were predominantly observed in the pyramidal neurons of Ammon’s horn in the hippocampus of both 5xFADXTg30 and 5xFADXTg30XAPP-/- mice (Figure 3) but were absent in the brains of WT and APP-/- mice (Figure S7). Quantitative analyses of pTau-positive neurons revealed a significant increase in both misfolded tau (Figure 3A–C) and hyperphosphorylated tau (Figure 3D–I) in the hippocampus of 5xFADXTg30XAPP-/- mice compared to 5xFADXTg30 controls. This observation was further confirmed by an analysis of the immunolabelled area, which showed a significant increase in MC1, PHF1, and AT8 immunoreactivity in the hippocampus of 5xFADXTg30XAPP-/- mice (Figure S8).

**Figure 4:**
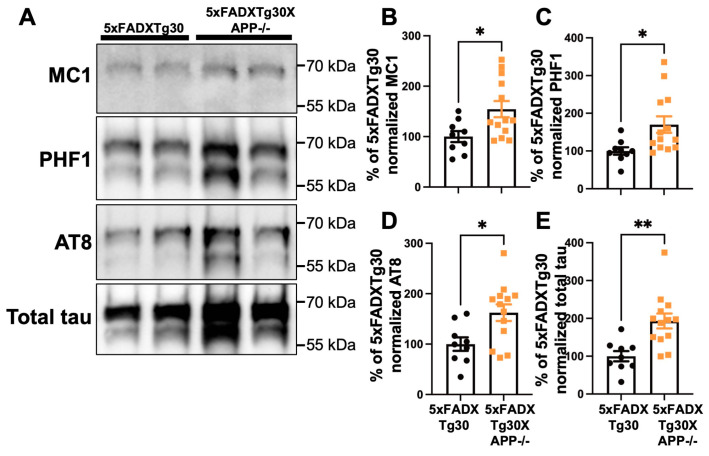
Murine APP deletion increased tau phosphorylation and aggregation in the brains of 5xFADXTg30XAPP-/- mice at 10 months. (**A**) WB analyses of sarkosyl-insoluble aggregated tau in the brains of 10-month-old mice. Sex-matched 10-month-old mouse brains were analyzed by WB (n = 9 for 5xFADXTg30 and n = 13 for 5xFADXTg30XAPP-/- mice). The sarkosyl-insoluble fraction was analyzed for MC1 (tau in pathological conformation), PHF1 (pSer396/Ser404 tau), AT8 (pSer202/Thr205 tau), and total tau (Dako A0024). (**B**–**E**) Quantification of sarkosyl-insoluble total tau levels. The levels of MC1 (**B**), PHF1 (**C**), AT8 (**D**), and total tau (**E**) were significantly increased in 5xFADXTg30XAPP-/- mice compared to 5xFADXTg30 mice. The optical density (OD) values in 5xFADXTg30 mice were used as the baseline (100%) and normalized to total protein concentration. Data are presented as mean ± SEM (* *p* < 0.05, ** *p* < 0.01 unpaired *t*-test). The original blots are shown in Figures S9 and S10.

To further explore whether APP deletion promotes tau aggregate formation in the brain, we analyzed the sarkosyl-insoluble fraction from the hemispheres of 5xFADXTg30 and 5xFADXTg30XAPP-/- mice by WB for the presence of pathological PHF-tau proteins (Figure 4A). A significant increase in misfolded (MC1), hyperphosphorylated (PHF1 and AT8), and total tau was observed in the sarkosyl-insoluble fraction of 5xFADXTg30XAPP-/- mice compared to 5xFADXTg30 controls (Figure 4B–E). These data suggest that murine APP deletion exacerbates tau pathology in 5xFADXTg30XAPP-/- mice compared to 5xFADXTg30 mice.

### 3.4. Amyloid Pathology Is Aggravated in the Hippocampus of 5xFADXTg30APP-/- Mice Compared to 5xFADXTg30 Mice

**Figure 5:**
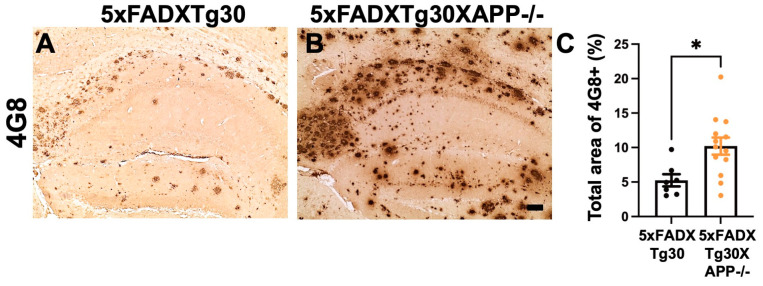
Amyloid pathology is aggravated in the hippocampus of 5xFADXTg30XAPP-/- mice compared to 5xFADXTg30 mice. (**A**,**B**) Representative immunostaining of Aβ using the 4G8 antibody in the hippocampus of 5xFADXTg30 (**A**) and 5xFADXTg30XAPP-/- (**B**) mice. (**C**) Quantification of 4G8-positive immunolabelling in the hippocampus. A significant increase in 4G8-positive structures was observed in the hippocampus of 5xFADXTg30XAPP-/- compared to 5xFADXTg30 controls. Ten-month-old male mice (5XFADXTg30: n = 7 and 5xFADXTg30XAPP-/-: n = 13) were analyzed. Data are presented as mean ± SEM (* *p* < 0.05 by unpaired *t*-tests). Scale bar, 60 µm.

To evaluate the effect of murine APP deletion on amyloid pathology progression, immunolabelling of 4G8-positive Aβ plaques was analyzed in the hippocampus (Figure 5A,B). The data suggested that murine APP deletion resulted in approximately 2-fold increase in 4G8-positive amyloid plaques in the hippocampus of 5xFADXTg30XAPP-/- mice compared to 5xFADXTg30 controls (Figure 5C).

### 3.5. Gliosis Is Aggravated in 5xFADXTg30XAPP-/- Mice Compared to 5xFADXTg30 Mice

**Figure 6:**
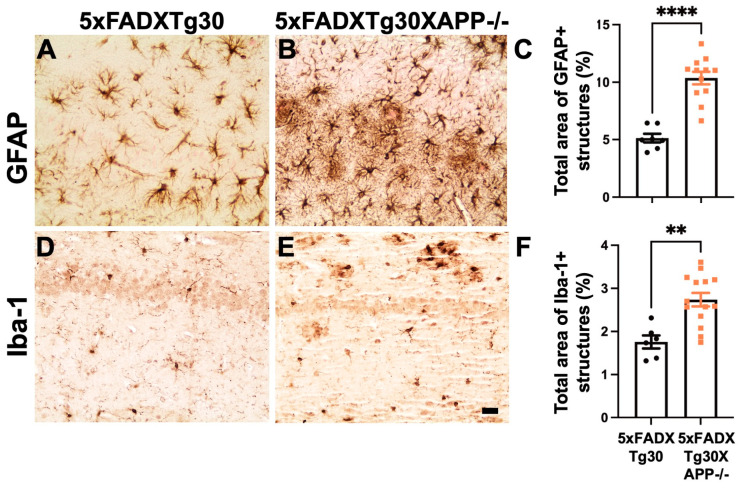
Gliosis is aggravated in 5xFADXTg30XAPP-/- mice compared to 5xFADXTg30 mice. (**A**–**F**) Representative immunostaining of the astrocyte marker GFAP (**A**,**B**) and the microglia marker Iba-1 (**D**,**E**) in the Ammon’s horn of the hippocampus of 5xFADXTg30 (**A**,**D**) and 5xFADXTg30XAPP-/- mice (**B**,**E**). Quantification of GFAP (**C**) and Iba-1 staining (**F**) showed a significant increase in both markers in the hippocampus of 5xFADXTg30XAPP-/- mice compared to 5xFADXTg30 mice. Ten-month-old male mice were analyzed (5XFADXTg30: n = 7 and 5xFADXTg30XAPP-/-: n = 13). Scale bar, 20 µm. Data are presented as mean ± SEM (** *p* < 0.01, **** *p* < 0.0001 by unpaired *t*-tests).

Our data support the hypothesis that the deletion of murine APP significantly impacts both tau and Aβ pathologies in the hippocampus of transgenic 5xFADXTg30 mice. These two pathologies are the main neuropathological features of AD and may trigger associated changes, such as gliosis, that are commonly observed in AD brains. To further investigate whether murine APP deletion influences gliosis in this context, we performed immunohistochemical analysis of astrocyte marker GFAP and microglia marker Iba-1 (Figure 6A–F). Quantitative analyses revealed a significant increase in both GFAP- and Iba-1-positive structures in the hippocampus of 5xFADXTg30XAPP-/- mice compared to 5xFADXTg30 mice.

These results collectively demonstrate a significant exacerbation of motor deficits, tau and Aβ pathologies, and gliosis in the brains of 5xFADXTg30XAPP-/- mice compared to 5xFADXTg30 mice.

## 4. Discussion

In this study, we demonstrate that deletion of murine APP exacerbates muscle weakness, motor deficits, AD lesions, and gliosis in the novel transgenic mouse line 5xFADXTg30XAPP-/- compared to 5xFADXTg30 mice. The absence of murine APP further worsened motor impairments, amyloid load, and tau pathologies induced by the expression of human mutant tau, APP, and PS1 in 5xFADXTg30 mice [32]. To our knowledge, this is the first experimental evidence that murine APP deletion leads to the exacerbation of both amyloid and tau pathologies in vivo.

Pathological tau in the sarkosyl-insoluble fraction was significantly elevated in the brains of 5xFADXTg30XAPP-/- mice compared to 5xFADXTg30 mice. Consistent with immunohistochemical results for MC1, AT8, and PHF1, tau pathology was significantly increased in the hippocampus of 5xFADXTg30XAPP-/- mice. Importantly, the exacerbation of tau pathology in these mice was not due to differences in tau expression, as total tau levels (human and murine) were comparable between 5xFADXTg30XAPP-/- and 5xFADXTg30 mice. This finding is consistent with the exacerbation of tau pathology observed in APPKOTg30 mice compared to Tg30 mice [15], suggesting a protective role for endogenous murine APP and its fragments in regulating tau pathology progression in vivo. Alternatively, the increased amyloid pathology observed in 5xFADXTg30XAPP-/- mice could also contribute the exacerbation of tau pathology, as amyloid-induced increases in tau pathology have been previously reported in 5xFADXTg30 mice compared to Tg30 mice [32].

While total APP expression (human and murine) was significantly reduced in 5xFADXTg30XAPP-/- mice due to the deletion of murine APP, amyloid pathology was notably increased in the hippocampus of these mice compared to 5xFADXTg30 controls. Previous studies have shown that deletion of endogenous murine APP resulted in a ~35% reduction in Aβ levels in the APP23 model but had no effect on Aβ levels in the APPPS1 model [33]. This discrepancy may arise from differences in the progression of amyloid pathology between the APP23 and 5xFAD models [34] or from the presence of tau pathology in our model.

The underlying mechanism driving the increased amyloid pathology in 5xFADXTg30XAPP-/- mice remains unclear. It is uncertain whether this increase is due to enhanced Aβ production, impaired Aβ clearance, or accelerated Aβ aggregation in the hippocampus. Notably, Mahler et al. reported that murine Aβ has a lower amyloidogenic potential compared to human Aβ [33], suggesting that the absence of less amyloidogenic murine Aβ may allow human Aβ to accumulate more extensively, contributing to the observed increase in amyloid deposits. Additionally, the absence of endogenous APP may lead to more amyloidogenic processing of mutant human APP due to the lack of competition between murine and mutant human APP.

To better understand the role of murine APP deletion in amyloid pathology progression, future studies focusing on the 5xFADXAPP-/- mouse line in an amyloid-only model (without tau pathology) could provide valuable insights. Although such studies are beyond the scope of this work, they will help delineate whether the observed increase in amyloid pathology in 5xFADXTg30XAPP-/- mice is driven by tau pathology or other mechanisms.

Mice with APP deletion exhibit motor deficits and muscle weakness [13,14]. Consistently, we observed an exacerbation of motor deficits in 5xFADXTg30XAPP-/- mice compared to 5xFADXTg30 mice. Importantly, the increased amyloid and tau pathologies observed in 5xFADXTg30XAPP-/- mice likely contribute to this exacerbation of motor deficits. Previous studies have shown that elevated tau pathology in the 5xFADXTg30 line similarly exacerbates motor deficits [32]. It is plausible that the deletion of murine APP acts as a sensibilizing factor, exacerbating the motor deficits caused by these pathologies.

Our study also suggests that gliosis, measured by both astrogliosis (GFAP-positive astrocytes) and microgliosis (Iba-1-ipositive microglia), is generally increased in 5xFADXTg30XAPP-/- mice compared to 5xFADXTg30 controls. These findings align with previous reports showing exacerbated astrogliosis in APP-/- mice compared to APP+/+ mice [13,35] and in APPKOTg30 mice compared to Tg30 controls [15]. Therefore, the aggravated astrogliosis observed in 5xFADXTg30XAPP-/- mice is likely, at least in part, a consequence of murine APP deletion. Nonetheless, we cannot exclude the possibility that exacerbated tau and amyloid pathologies in these mice further contribute to increased gliosis, as both pathologies are closely linked to astrocytic activation [16,17].

Regarding microgliosis, a previous study found no significant change in Iba-1-positive microglia in APPKOTg30 mice compared to Tg30 mice [15]. Given that tau pathology was exacerbated in APPKOTg30 mice in the absence of amyloid pathology, the increased microgliosis observed in 5xFADXTg30XAPP-/- mice is likely driven by the elevated amyloid load in these animals.

APP is cleaved by α- or β-secretases to release its metabolites with critical roles in cellular functions. For instance, Aβ plays a critical role in maintaining normal synaptic function. Reduction in endogenous Aβ through genetic or pharmaceutical inhibition leads to disturbances in synaptic morphology, plasticity, and cognitive function [36]. Moreover, the N-terminal soluble fragments sAPPα and sAPPβ are produced. Notably, sAPPα is 100-fold more neurotrophic than sAPPβ [37]. Interestingly, the level of sAPPα is decreased in the cerebrospinal fluid and platelets of Alzheimer’s patients at early stages and is inversely correlated with cognitive decline [38,39,40,41,42]. Several independent studies have reported the neurotrophic effects of sAPPα in mouse brains [4,43,44]. For example, sAPPα overexpression has been shown to inhibit key tau kinases such as GSK3β [45] and CDK5 [46,47] in various models. A recent study indicated that adeno-associated virus (AAV)-mediated overexpression of sAPPα partially rescued tau pathology and deficits in spine density in the tau transgenic mouse model THY-Tau22 [48].

sAPPα is abundant in wild-type, non-transgenic mouse brains due to the physiologically predominant non-amyloidogenic APP processing pathway [49], but it is absent in APP-/- mouse brains. The human mutant APP expressed in the 5xFAD model carries several mutations, including the Swedish mutation (K670N and M671L), which is located at the β-secretase cleavage site of APP [50] and promotes cleavage by β-secretases [51]. Given this, the level of sAPPα is expected to be severely reduced in the brains of 5xFADXTg30XAPP-/- mice. This reduction may contribute to the significant aggravation of AD phenotypes observed in these mice compared to 5xFADXTg30 controls. Further studies to test adeno-associated virus (AAV)-mediated overexpression of sAPPα in 5xFAD×Tg30×APP-/- mice should be informative to determine whether this intervention can ameliorate tau pathology, amyloid load, and gliosis. Such an approach would provide valuable information of the function of sAPPα.

We acknowledge several limitations in this study. First, the sample size was heterogeneous in the 5xFADXTg30 and 5xFADXTg30XAPP-/- groups due to a limited number of sex-matched samples. This limitation may have introduced variability in our results. Second, although we observed that murine APP deletion exacerbates tau pathology, amyloid load, and gliosis in the hippocampus of 5xFADXTg30XAPP-/- mice compared to 5xFADXTg30 mice, the precise mechanism underlying these observations remains unclear. Further studies are needed to understand exactly how murine APP is involved in the pathogenesis of amyloid and tau pathologies, as well as the associated gliosis. Third, whereas the 5xFAD×Tg30×APP-/- line provides a powerful AD model for determining the effects of murine APP deletion on both amyloid and tau pathologies, it is based on a highly specific transgenic context, including mutant human APP, PS1, and tau overexpression. Therefore, the direct relevance of findings to human AD requires careful consideration. Despite these limitations, the 5xFAD×Tg30×APP-/- model remains a powerful tool for exploring the role of murine APP and its derivatives in amyloid and tau pathologies, as well as gliosis.

## 5. Conclusions

In conclusion, the novel 5xFADXTg30XAPP-/- mouse model, which exhibits both amyloid and tau pathologies, provides a valuable tool for investigating the roles of murine APP and its derivatives in AD lesions, including amyloid and tau pathologies, as well as astrogliosis and microgliosis. Our study supports the hypothesis that APP and its fragments may play a key role in mitigating tau aggregation, amyloid pathology, and gliosis.

## Data Availability

The original contributions presented in this study are included in the article/supplementary material. Further inquiries can be directed to the corresponding authors. Full-length uncropped WB images are available in Supplementary figures.

## Supplementary Materials

The following supporting information can be downloaded at: https://www.mdpi.com/article/10.3390/biom15020159/s1, Figure S1: Summary of the fractionation protocol used to obtain RIPA-soluble and sarkosyl insoluble fractions. Figures S2–S5: Full-length blots for the cropped images shown in the Figure 1A. Figure S6: Grip test and rotarod test in WT and APP-/- mice. Figure S7: The representative images of the immunohistochemistry for MC1, PHF1, and AT8 in the hippocampus of WT and APP-/- mice. Figure S8: Quantification of total area immunolabelled by MC1, PHF1, and AT8. Figures S9 and S10: Full-length blots for the cropped images shown in the Figure 4A.

## Abbreviations

AAV	adeno-associated-virus
Aβ	amyloid β
AD	Alzheimer’s disease
AICD	amyloid precursor protein intracellular domain
APP	amyloid precursor protein
CTF	C-terminal fragment
FAD	familial AD
FTLD	frontotemporal lobar degeneration
GFAP	Glial Fibrillary Acidic Protein
LTP	Long-term potential
NFTs	neurofibrillary tangles
PBS	phosphate buffer saline
PHF-tau	paired-helical filament
PS1	presenilin-1
sAPPα	soluble N-terminal APPα
sAPPβ	soluble N-terminal APPβ
WB	Western blot
