# Supplementary material for: Deletion of Murine APP Aggravates Tau and Amyloid Pathologies in the 5xFADXTg30 Alzheimer’s Disease Model
Source: Biomolecules. 2025 Jan 21;15(2):159. doi: 10.3390/biom15020159 (PMC11853399; doi:10.3390/biom15020159)
Supplement: Supplementary file 1 [file biomolecules-15-00159-s001.zip › biomolecules-3386894-supplementary.pdf]

Supplementary information

Deletion of murine APP aggravates tau and amyloid pathologies in an Alzheimer's disease model of 5xFADXTg30

Kunie Ando[1*], Andreea-Claudia Kosa[1*], Yasmina Mehadji[1*], Hinde Lasri[1], Lidia Lopez-Gutierrez[1], Carolina Quintanilla-Sánchez[1], Emmanuel Aydin[1], Emilie Doeraene[1], Alain Wathelet-Depauw[1], Siranjeevi Nagaraj[1], Jean-Pierre Brion[1], Karelle Leroy[1]

[1] Alzheimer and other tauopathies research group, ULB Center for Diabetes Research, Medical Faculty, Université Libre de Bruxelles, ULB Neuroscience Institute, 808 route de Lennik, B-1070 Brussels, Belgium.

***Authors equally contributed**

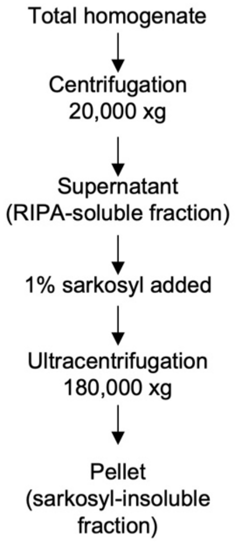

Supplementary Fig. S1

Summary of the fractionation protocol used to obtain RIPA-soluble and sarkosyl insoluble fractions.

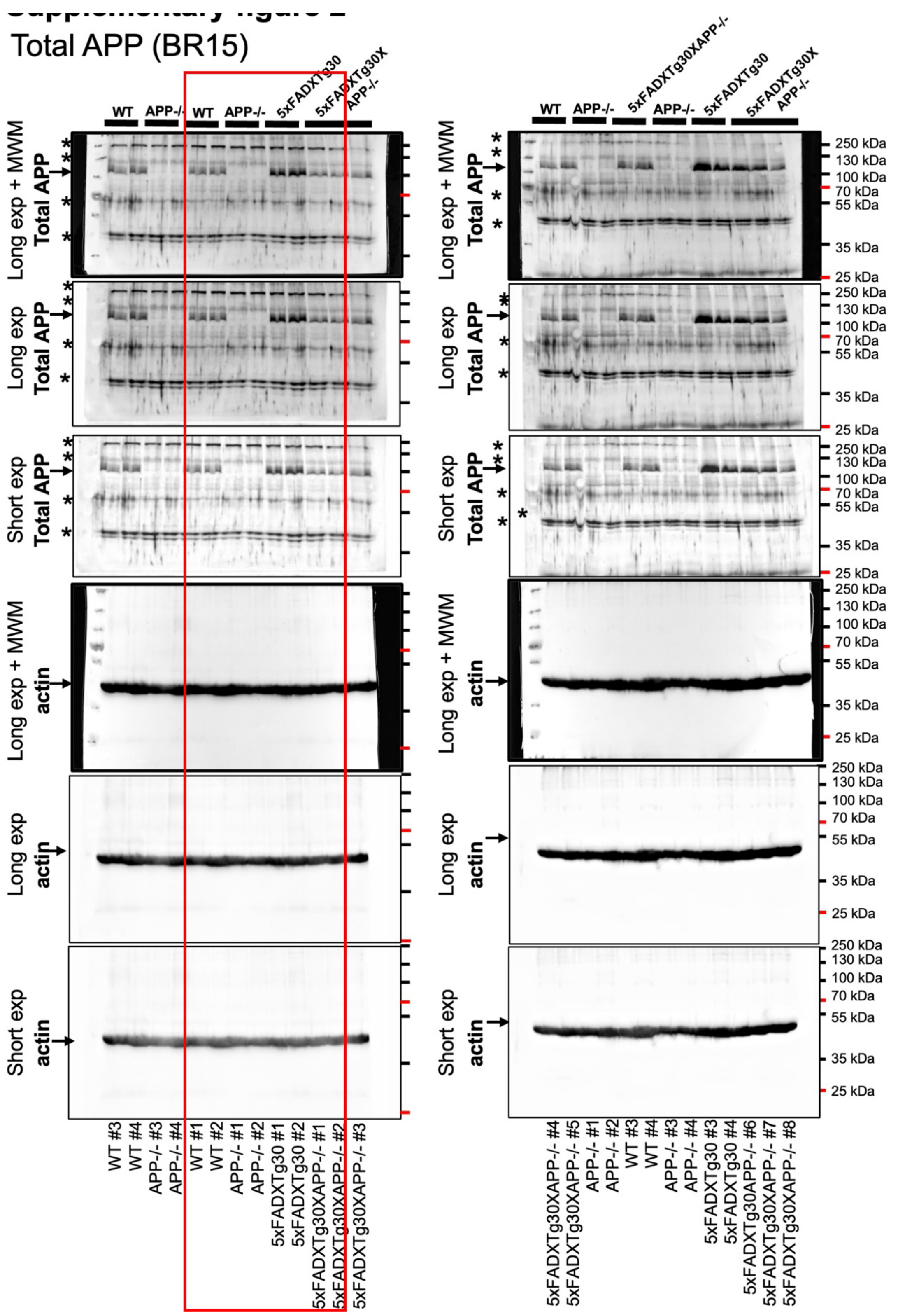

Supplementary figure S2

Full-length blots corresponding to the cropped images of total APP (detected using the BR15 antibody) shown in Figure 1A. Chemiluminescence signals from the long exposure are merged with the molecular weight marker (MWM). Arrows indicate the specific band, while the asterisks denote non-specific signals. The red square highlights the representative result shown in Figure 1A. "Exp" denotes exposure time.

Human APP (3H5)

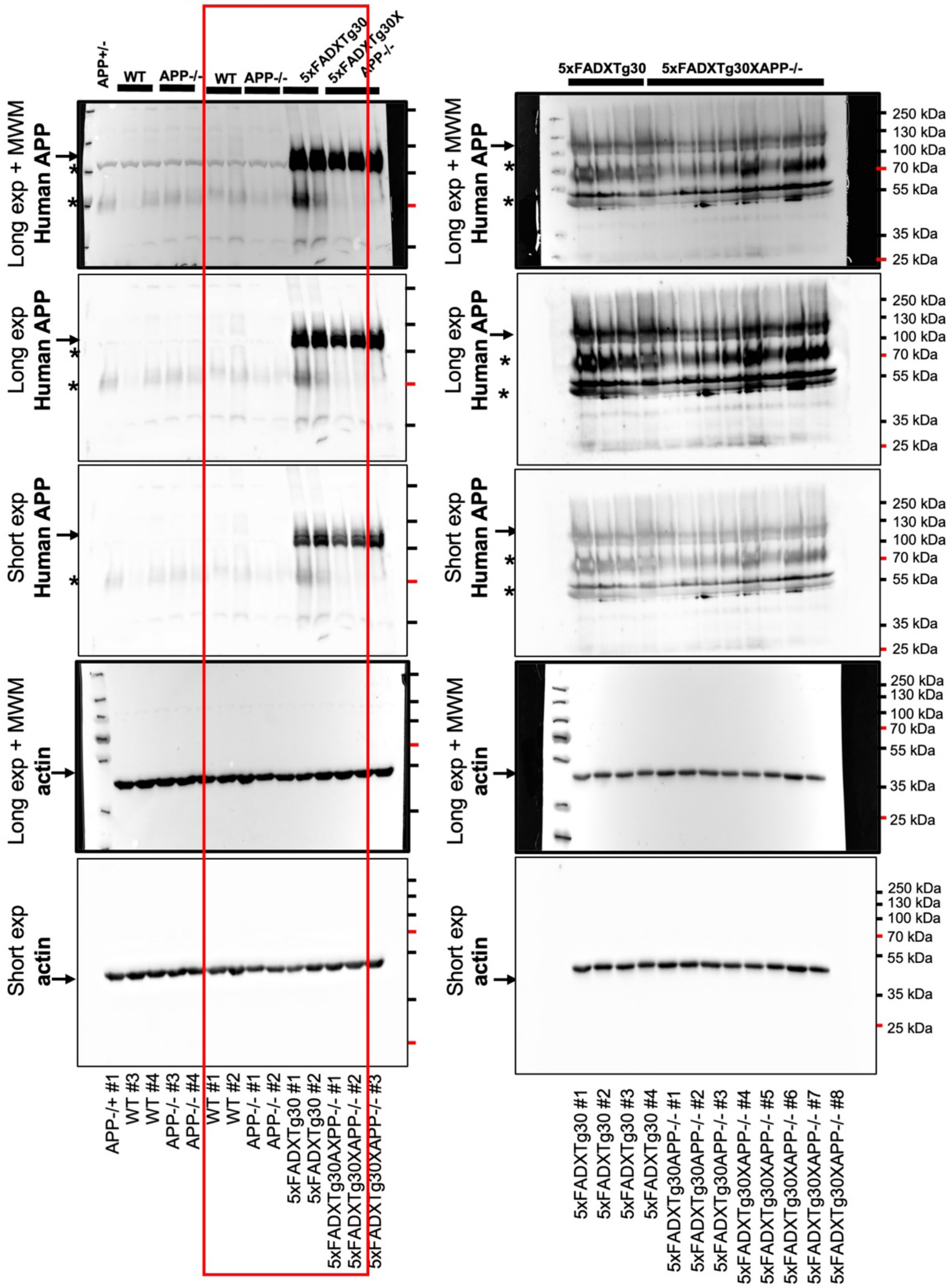

Supplementary figure S3

Full-length blots corresponding to the cropped images of human APP (detected using the 3H5 antibody) shown in Figure 1A. Chemiluminescence signals from the long exposure are merged with the molecular weight marker (MWM). Arrows indicate the specific band, while the asterisks denote non-specific signals. The red square highlights the representative result shown in Figure 1A. “Exp” denotes exposure time.

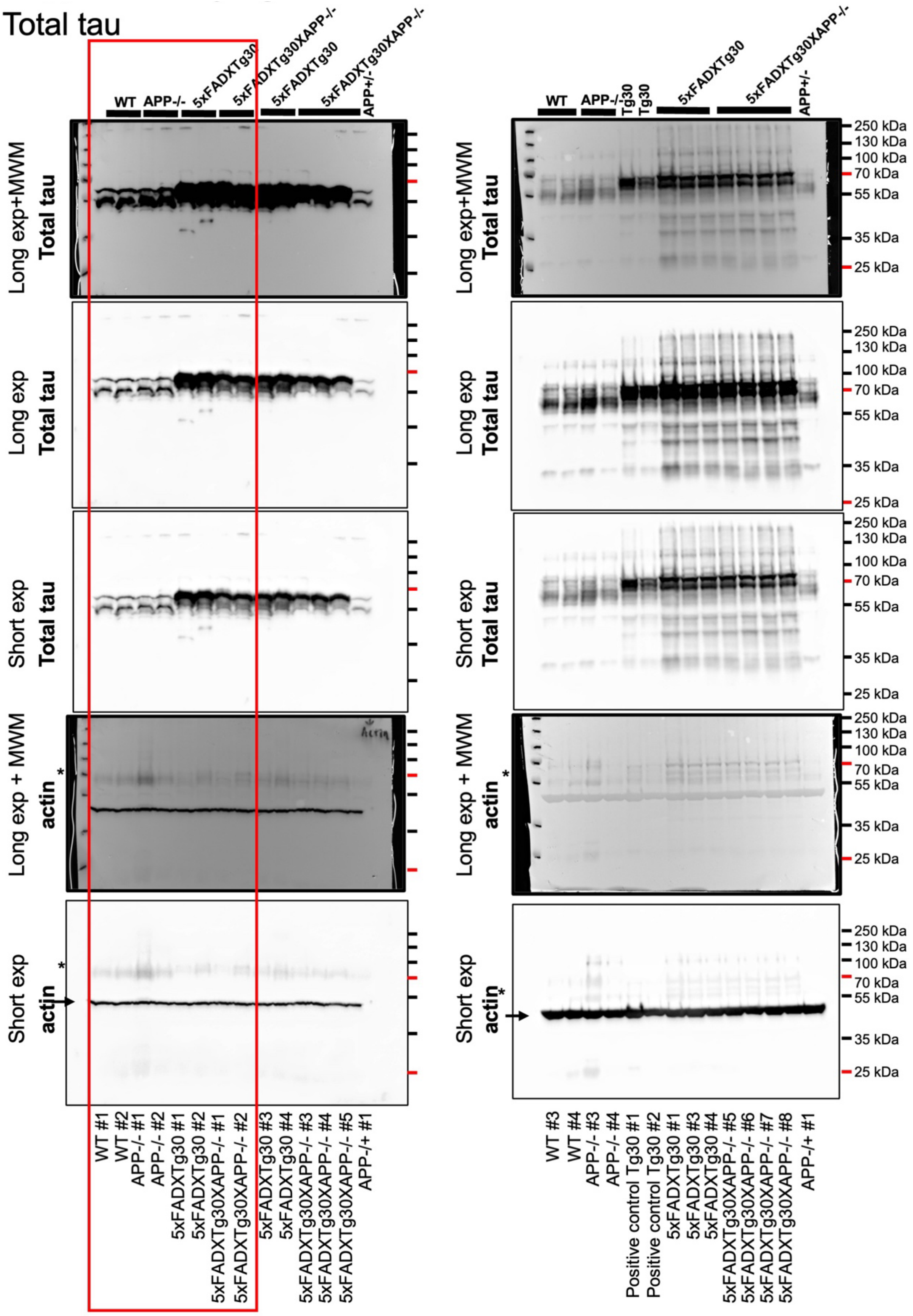

Supplementary figure S4

Full-length blots corresponding to the cropped images of total tau (detected by the Dako A0024 antibody) shown in Figure 1A. Chemiluminescence signals from the long exposure are merged with the molecular weight marker (MWM). Arrows indicate the specific band, while the asterisks denote non-specific signals. The red square highlights the representative result shown in Figure 1A. “Exp” denotes exposure time.

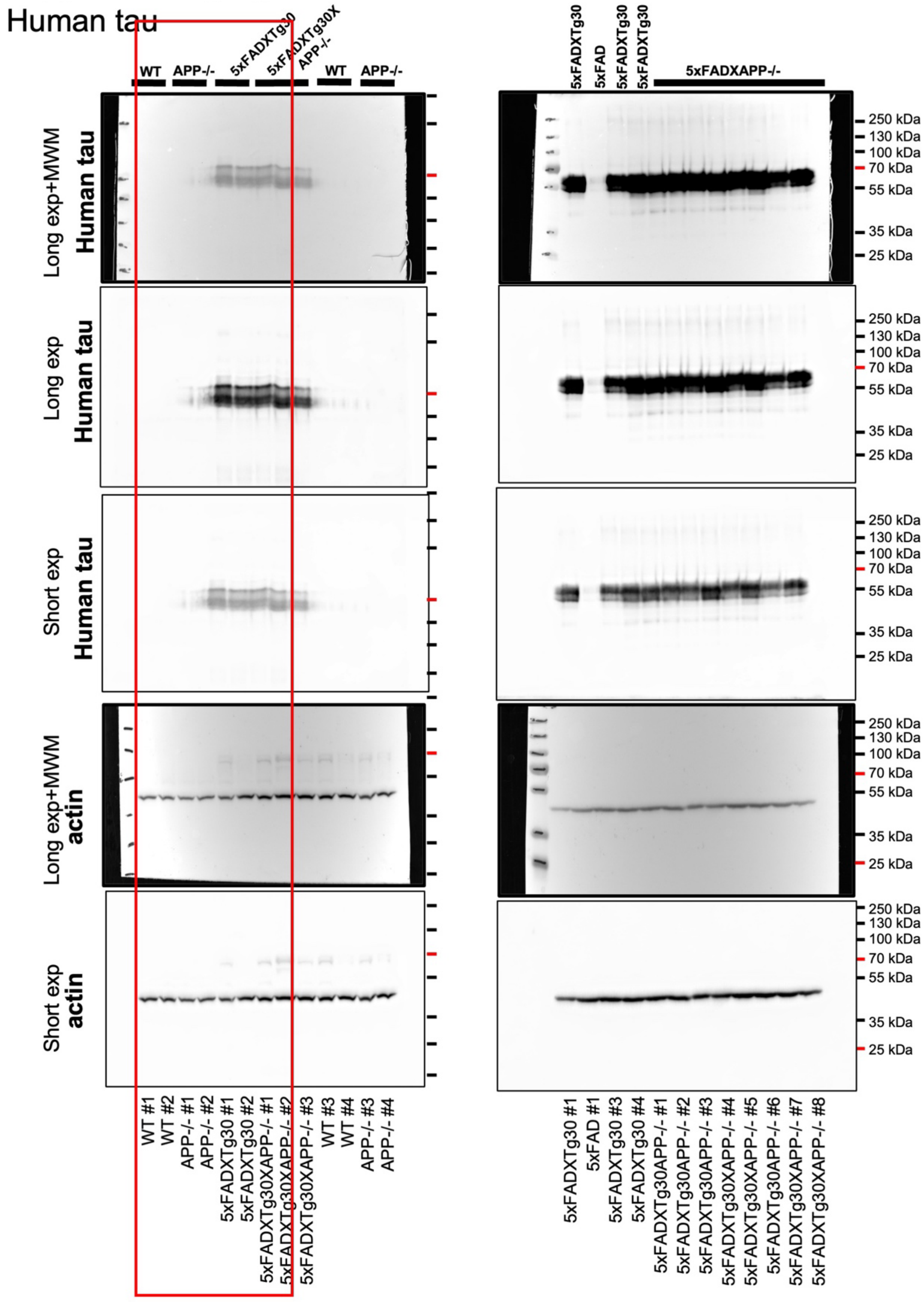

Supplementary figure S5

Full-length blots corresponding to the cropped images of human tau (detected by the BR21 antibody) shown in Figure 1A. Chemiluminescence signals from the long exposure are merged with the molecular weight marker (MWM). The brain lysate of 5xFADXTg30 #2 was unavailable for the WB analysis shown in the right panel and was replaced with a negative control (5xFAD #1). Arrows indicate the specific band, while the asterisks denote non-specific signals. The red square highlights the representative result shown in Figure 1A. “Exp” denotes exposure time.

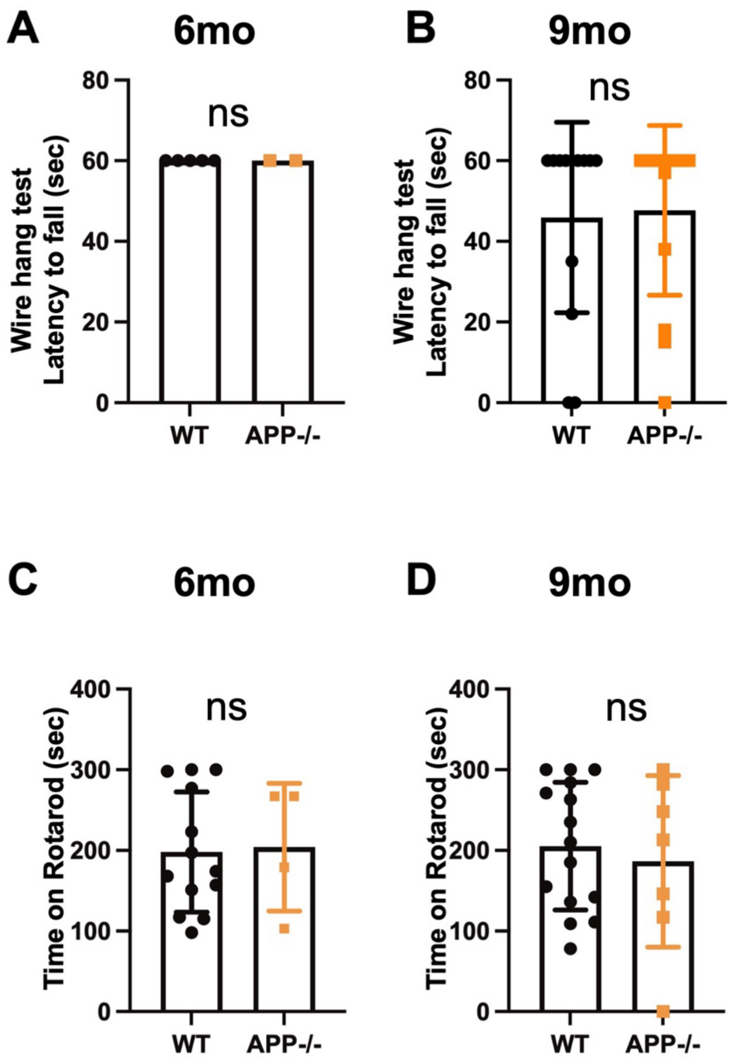

Supplementary Fig. S6

No significant difference was observed in motor phenotypes of APP-/- mice compared to WT littermates at 6 and 9 months old.

(**A-B**) Performance on the wire hang test shown as the latency to fall from a suspended grid was not significantly altered in APP-/- mice at 6 months, (A, n=5 for WT and n=2 APP-/-) or at 9 months (B, n=13 and n=14, respectively).

(**C-D**) Performance on the accelerated rotarod was not significantly different between WT and APP-/- at 6 months (C, n=13 and n=4, respectively or at 9 months (D, n=15 and n=7, respectively). Data are presented as mean ± SEM by student t-test. Ns, not significant.

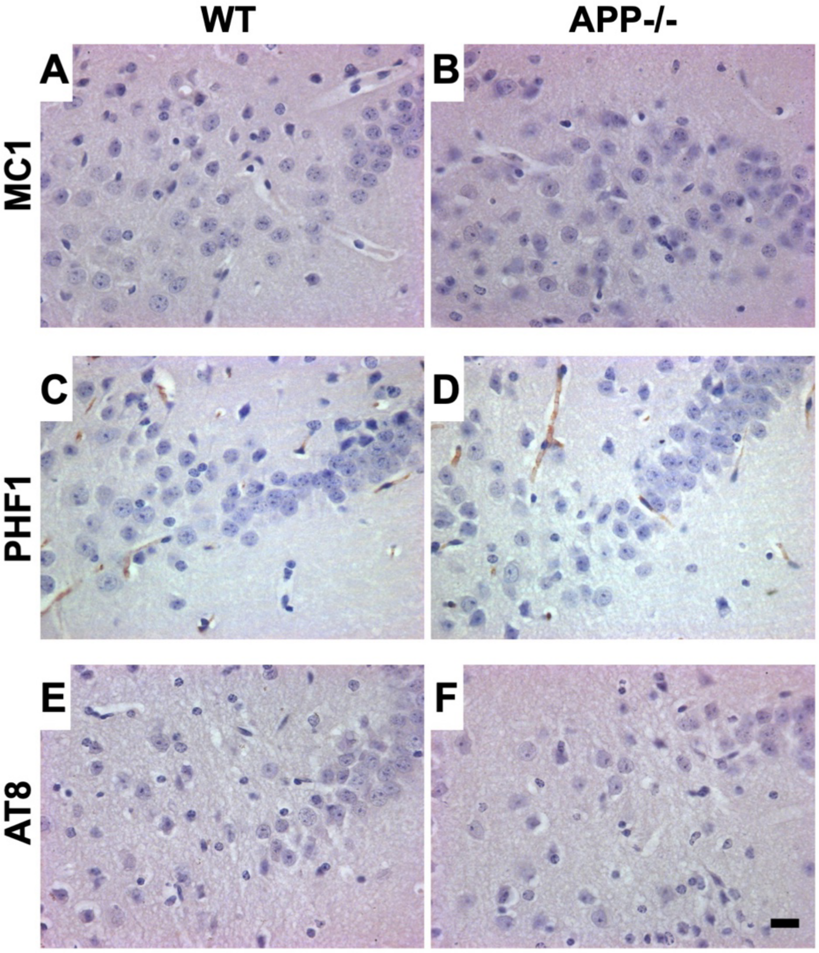

Supplementary Fig. S7

No staining of pTau was detected in the hippocampus of WT or APP-/- mice at 10 months old. (**A-F**) Representative immunostaining for pathological tau using MC1 (**A, B**), PHF1 (**C, D**) and AT8 antibodies (**E, F**) in the CA1 pyramidal neurons of the Ammon's horn of the hippocampus from WT and APP-/- mice. There was no significant staining of misfolded tau (MC1) and hyperphosphorylated tau (pSer202/Thr205 for AT8 and pSer396/Ser404 for PHF1) in WT and APP-/- mice. Analysis was performed on 10-month-old male mice (WT: n=2 and APP-/- n=2). Haematoxylin counterstaining shows the nuclei in blue. Scale bar, 20 µm.

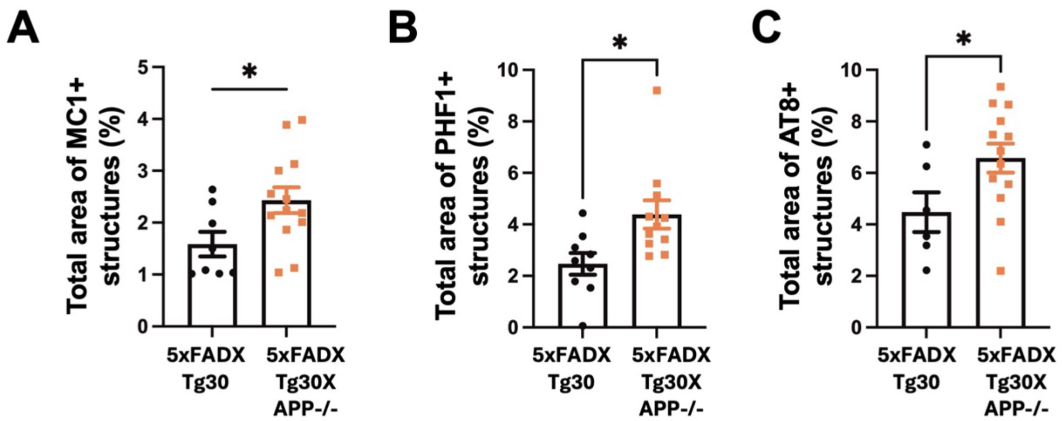

Supplementary Fig. S8

Increased progression of tau pathology in the hippocampus of 5xFADXTg30XAPP-/- mice compared to 5xFADXTg30.

(**A-C**) Quantification of the immunolabelled area in the hippocampus for misfolded tau (MC1) and hyperphosphorylated tau (pSer396/Ser404 for PHF1 and pSer202/Thr205 for AT8) in 5xFADXTg30XAPP-/- mice compared to 5xFADXTg30 mice. Analysis was performed on 10-month-old male mice (5XFADXTg30: n=6-8 and 5xFADXTg30XAPP-/- n=11-13). Data are presented as mean ± SEM (*$p < 0.05$ by unpaired t-tests).

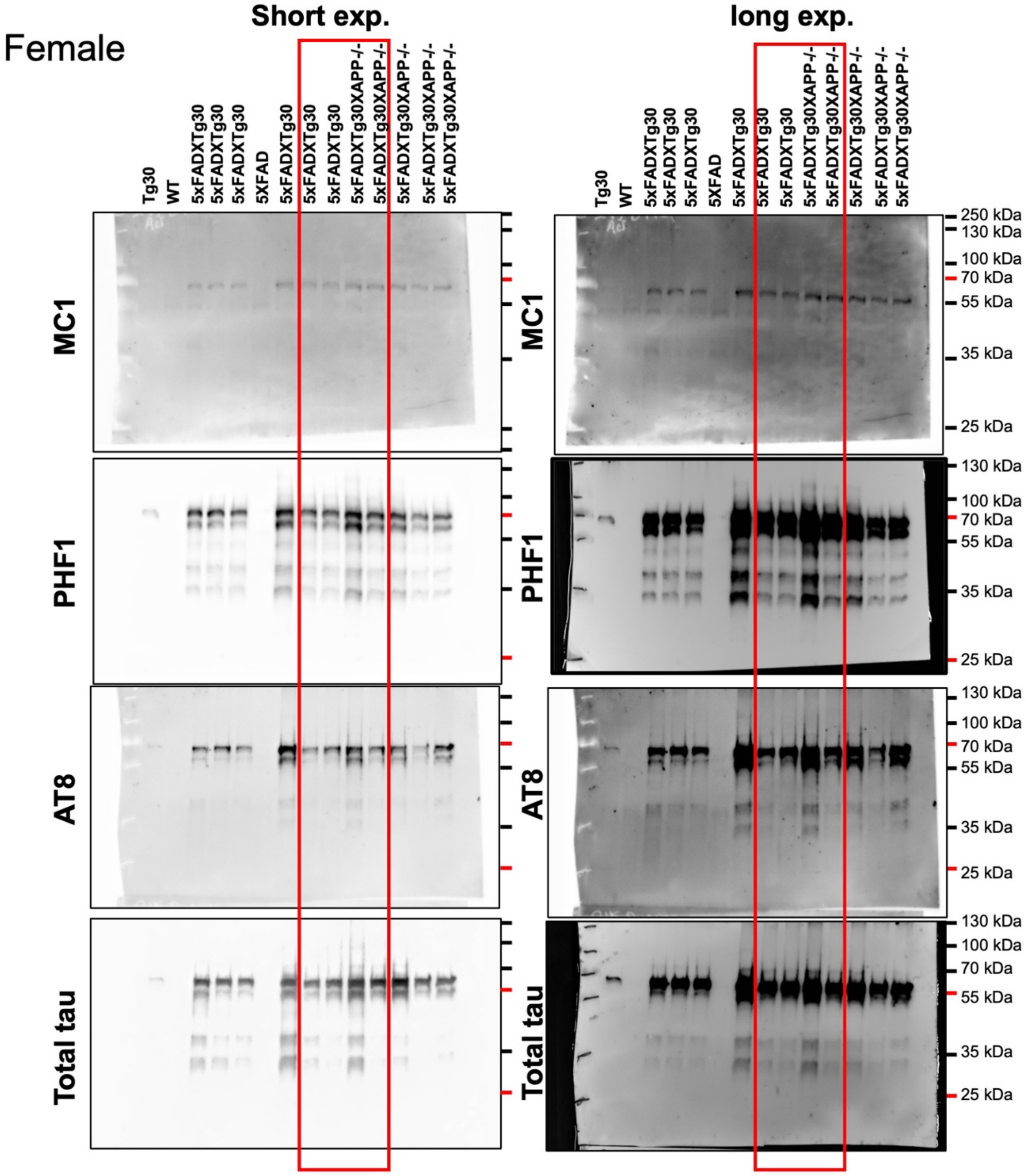

Supplementary Figure S9

Full-length blots corresponding to the cropped images shown in Figure 4A (left column), representing analyses of sarkosyl-insoluble tau from female 5xFADXTg30 and 5xFADXTg30XAPP-/- mice. The red square highlights the representative result shown in Figure 4A. To indicate molecular weight, chemiluminescence signals from long exposure or merged images with blot markers are shown in the right panels. Positive control (Tg30) and negative controls (WT and 5xFAD) were included. "Exp" denotes exposure time.

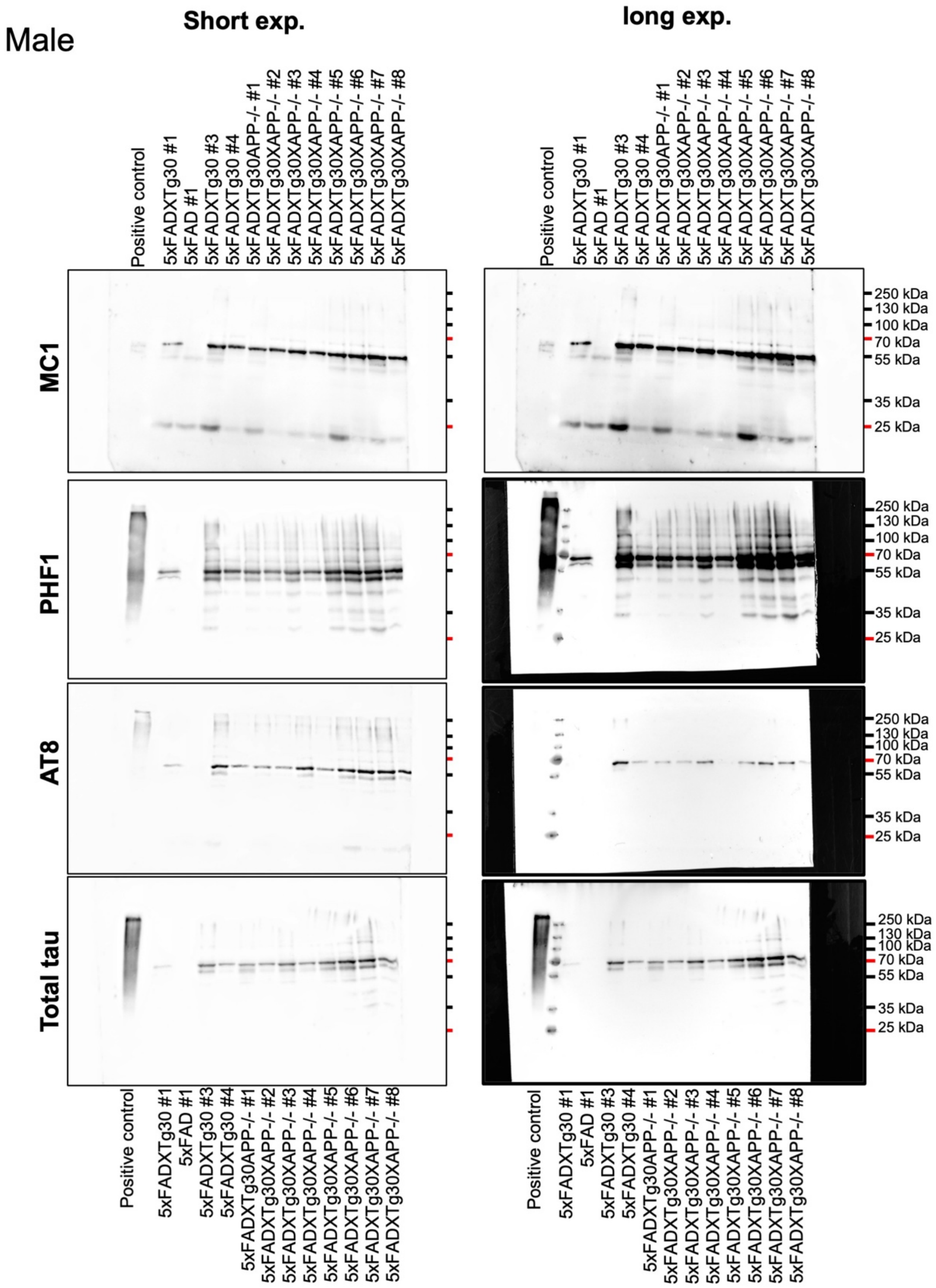

Supplementary figure S10

Full-length blots of sarkosyl-insoluble tau obtained analyses male 5xFADXTg30 and 5xFADXTg30XAPP-/-. The brain lysate of 5xFADXTg30 #2 was unavailable for this analysis and was replaced with a negative control (5xFAD #1). To indicate molecular weight, chemiluminescence signals from long exposure or merged images with blot markers are shown in the right panels. “Exp” denotes exposure time.
